# Neuropathological Mechanisms of Seizures in Autism Spectrum Disorder

**DOI:** 10.3389/fnins.2016.00192

**Published:** 2016-05-10

**Authors:** Richard E. Frye, Manuel F. Casanova, S. Hossein Fatemi, Timothy D. Folsom, Teri J. Reutiman, Gregory L. Brown, Stephen M. Edelson, John C. Slattery, James B. Adams

**Affiliations:** ^1^Autism Research Program, Arkansas Children's Research InstituteLittle Rock, AR, USA; ^2^Department of Pediatrics, University of Arkansas for Medical SciencesLittle Rock, AR, USA; ^3^Department of Biomedical Sciences, University of South Carolina School of Medicine GreenvilleGreenville, SC, USA; ^4^Department of Psychiatry, University of Minnesota Medical SchoolMinneapolis, MN, USA; ^5^Serenity Health Care CenterWaukesha, WI, USA; ^6^Autism Research InstituteSan Diego, CA, USA; ^7^School for Engineering of Matter, Transport, and Energy, Arizona State UniversityTempe, AZ, USA

**Keywords:** autism spectrum disorder, seizures, epilepsy, genetic syndrome, metabolic disorders, excitatory-to-inhibitory cortical balance, gamma-aminobutyric acid

## Abstract

This manuscript reviews biological abnormalities shared by autism spectrum disorder (ASD) and epilepsy. Two neuropathological findings are shared by ASD and epilepsy: abnormalities in minicolumn architecture and γ-aminobutyric acid (GABA) neurotransmission. The peripheral neuropil, which is the region that contains the inhibition circuits of the minicolumns, has been found to be decreased in the post-mortem ASD brain. ASD and epilepsy are associated with inhibitory GABA neurotransmission abnormalities including reduced GABA_A_ and GABA_B_ subunit expression. These abnormalities can elevate the excitation-to-inhibition balance, resulting in hyperexcitablity of the cortex and, in turn, increase the risk of seizures. Medical abnormalities associated with both epilepsy and ASD are discussed. These include specific genetic syndromes, specific metabolic disorders including disorders of energy metabolism and GABA and glutamate neurotransmission, mineral and vitamin deficiencies, heavy metal exposures and immune dysfunction. Many of these medical abnormalities can result in an elevation of the excitatory-to-inhibitory balance. Fragile X is linked to dysfunction of the mGluR5 receptor and Fragile X, Angelman and Rett syndromes are linked to a reduction in GABA_A_ receptor expression. Defects in energy metabolism can reduce GABA interneuron function. Both pyridoxine dependent seizures and succinic semialdehyde dehydrogenase deficiency cause GABA deficiencies while urea cycle defects and phenylketonuria cause abnormalities in glutamate neurotransmission. Mineral deficiencies can cause glutamate and GABA neurotransmission abnormalities and heavy metals can cause mitochondrial dysfunction which disrupts GABA metabolism. Thus, both ASD and epilepsy are associated with similar abnormalities that may alter the excitatory-to-inhibitory balance of the cortex. These parallels may explain the high prevalence of epilepsy in ASD and the elevated prevalence of ASD features in individuals with epilepsy.

## Introduction

Autism spectrum disorders (ASD) is a behaviorally defined disorder that has recently been estimated to affect as many as 1 out of 45 individuals (Zablotsky et al., 2015). Although, ASD is defined by behavioral features, it is associated with co-occurring medical conditions. For example, epilepsy is more prevalent in ASD than in the typically developing children with a prevalence ranging from 5 to 38% (Deykin and Macmahon, 1979; Volkmar and Nelson, 1990; Tuchman and Rapin, 2002; Danielsson et al., 2005; Hara, 2007). Data from surveys performed by the Autism Research Institute on over 1200 participants suggests that the prevalence is between 15 and 19%. Epilepsy is one of the most disabling ASD co-morbidities as children with ASD and epilepsy are more likely to have intellectual disability (Tuchman, 2013) and increased mortality (Shavelle et al., 2001; Pickett et al., 2011) as compared to children with ASD without epilepsy. In addition, epilepsy in ASD tends to be more treatment-resistant as compared to epilepsy in typically developing children (Sansa et al., 2011).

One of the major questions in ASD research is its etiology. Much ASD research concentrates on genetic causes (Rossignol and Frye, 2012b) even though inherited single gene and chromosomal defects only account for a minority of ASD cases (Schaefer et al., 2013). However, genetic etiologies may be overrepresented in children with ASD and epilepsy as many genetic syndromes and gene mutations associated with ASD include epilepsy as a common feature (Murdoch and State, 2013; Tuchman et al., 2013).

Although some have suggested that clinical seizures do not have any special causative significance in ASD (Tuchman and Rapin, 1997), ASD coexists with epilepsy in several disorders (see Section Specific Medical Disorders Associated with Both ASD and Epilepsy) suggesting that the same neuropathology may result in both ASD and epilepsy. Thus, this manuscript reviews the shared biological abnormalities in ASD and epilepsy in two sections. The section called Basic Neuropathological Mechanisms of Seizures in ASD discusses two neuropathological mechanisms that have been described in ASD that can also cause epilepsy. Both mechanisms involve an abnormal reduction in inhibitory mechanisms of the brain, thereby resulting in an increase in the excitatory-to-inhibitory balance. The section called Specific Medical Disorders Associated with Both ASD and Epilepsy will review specific clinical disorders that have been described in both ASD and epilepsy with special reference to underlying neuropathological mechanisms that can cause seizures.

Overall, our review finds that many disorders associated with ASD increase the excitatory-to-inhibitory balance by either (1) reducing inhibitory circuits in the brain through a decrease in the inhibitory neurotransmitter γ-aminobutyric acid (GABA), or (2) increasing excitatory circuits in the brain through an increase in glutamate neurotransmission. Elevation in the excitatory-to-inhibitory balance in the brain can lead to seizures. By carefully outlining these disorders, insight into the etiologies that underlie ASD may be better understood.

## Basic neuropathological mechanisms of seizures in ASD

Several neuropathological processes associated with ASD are also associated with epilepsy. Here we review two such neuropathological processes: (1) minicolumn architecture and (2) GABA neurotransmission.

### Minicolumn architecture

The minicolumn is a radially-oriented assembly of neurons and cellular elements considered to be an elemental modular microcircuit of the neocortex (Buxhoeveden and Casanova, 2002; Casanova et al., 2006). The minicolumn core contains pyramidal cell arrays surrounded by a peripheral neuropil space that contains GABAergic inhibitory interneurons and other cells such as the double-bouquet cell (Mountcastle, 1997; Buxhoeveden and Casanova, 2002; Defelipe, 2005). Double-bouquet cells feature axonal bundles which provide a vertical stream of inhibition (Mountcastle, 1997). This inhibitory stream insulates the minicolumn core from the excitation from other surrounding minicolumns (Defelipe et al., 1990; Favorov and Kelly, 1994; Defelipe, 1999).

The peripheral neuropil space has been shown to be reduced in post-mortem brain tissue from ASD individuals (Buxhoeveden and Casanova, 2002), with this reduction most prominent over the prefrontal cortex (Casanova et al., 2006). The neuropil space is reduced within the region that contains the inhibition circuits of minicolumns (Defelipe et al., 1990; Favorov and Kelly, 1994; Defelipe, 1999). These architectural changes should, theoretically, disrupt the normal balance between excitation and inhibition influences within the columnar organization of the cortex (Casanova et al., 2003). A reduction of GABAergic inhibitory activity has been proposed to result in hyperexcitability of minicolumn circuits and can explain some of the symptomatology observed in ASD, including the high incidence of seizures and auditory-tactile hypersensitivity (Rubenstein and Merzenich, 2003). Networks of inhibitory interneurons acting as GABA gated pacemakers are also critically involved in gamma oscillations (Grothe and Klump, 2000). Abnormalities in gamma oscillations are associated with problems with binding and the coactivation of neural assemblies. A deficit in binding and gamma oscillations has been proposed to explain many of the symptoms related to ASD (e.g., visuoperceptual defects, understanding and using context; Grice et al., 2001; Brock et al., 2002; Brown et al., 2005; Rippon et al., 2007; Tommerdahl et al., 2007).

### GABA transmission

GABA is the major inhibitory neurotransmitter of the central nervous system (CNS). Abnormalities in GABA neurotransmission have been associated with epilepsy. GABBR1A, GABBR1B, and GABBR2 receptor subunits are reduced in the hippocampi of patients with temporal lobe epilepsy (Princivalle et al., 2003), and animal models have also shown a link between GABA receptor expression and epilepsy (Schuler et al., 2001; Han et al., 2006). Individuals with ASD have been shown to have abnormalities in GABAergic brain systems (Blatt et al., 2001; Dhossche et al., 2002; Fatemi, 2008), as well as a reduction in GABA_A_(Fatemi et al., 2009) and GABA_B_ (Fatemi et al., 2009) receptor subunits in both the frontal and parietal cortices, as compared to controls, with the ASD group also demonstrating a markedly higher rate of epilepsy than the controls. In addition, the GABA subunits found to be reduced in individuals with ASD (i.e., GABRα1 and GABBR1) have been associated with childhood absence epilepsy, juvenile myoclonic epilepsy, and atypical absence seizures (Delgado-Escueta, 2007; Kang et al., 2009).

## Specific medical disorders associated with both ASD and epilepsy

### Genetic disorders

The neurobiological mechanisms leading to seizures in genetic syndromes that are associated with ASD are diverse and complex. Imbalances in GABA and glutamate have been suggested to underlie CNS dysfunction in several of these genetic syndromes. Defects in GABA_A_ function has been implicated in Fragile X (D'hulst and Kooy, 2007) and recent studies on Fragile X suggest that mGluR5 dysfunction results in heightened excitability and secondary alterations in GABA function (Frye, 2014). Dysfunction in GABA_A_ receptor function has also been implicated in Angelman syndrome (Pelc et al., 2008). Indeed, a cluster of genes coding for three GABA_A_ receptor subunits lie adjacent to the critical Angelman region (i.e., UBE3A). Mutations within the Rett syndrome gene (i.e., MECP2) decreases expression of GABRB3, a gene responsible for encoding the beta_3_ subunit of the GABA_A_ receptor, and DLX5, a gene which regulates the production of enzymes responsible for GABA production.

Some genetic syndromes associated with epilepsy and ASD are associated with metabolic abnormalities. For example, mouse models of both Angelman and Rett syndromes demonstrate mitochondrial dysfunction (Kriaucionis et al., 2006; Su et al., 2011) and mitochondrial dysfunction is reported in a Rett syndrome case (Condie et al., 2010). Phelan-McDermid Syndrome (PMS) and duplication of the 22q13 region are both associated with ASD and mitochondrial dysfunction (Frye, 2012b; Frye et al., 2016a). As mentioned below, disruption of mitochondrial metabolism can result in changes to the excitatory-to-inhibitory balance.

Single gene disorders associated with both ASD and epilepsy have been associated with abnormalities in the excitatory- to-inhibitory balance (Srivastava and Schwartz, 2014). Mutations in CNTNAP2 (Peñagarikano et al., 2011) or CNTNAP4 (Karayannis et al., 2014) result in reduced GABAergic neurotransmission. The SYNGAP1 haploinsufficiency animal model shows an increase in neuronal excitability and an increase in seizure susceptibility (Clement et al., 2012). Other genes are associated with a relative decrease in the excitatory-to-inhibitory balance. Animal model with NLGN3 mutations demonstrates increased inhibitory neurotransmission (Tabuchi et al., 2007). Cellular (Shcheglovitov et al., 2013) and animal models (Bangash et al., 2011; Wang et al., 2011b) demonstrate a reduction in excitatory neurotransmission when SHANK3 is disrupted. Animal models with decreased synapsin I (SYN1) demonstrate reduced glutamate release (Li et al., 1995).

### Metabolic disorders

#### Disorders of energy metabolism

Disorders of energy metabolism have been associated with ASD (Giulivi et al., 2010; Frye and Naviaux, 2011; Frye, 2012c; Rose et al., 2014a,b) and epilepsy (Frye, 2015). Some children with ASD have mitochondrial dysfunction that is different than classic mitochondrial disease (Frye and Rossignol, 2011; Rossignol and Frye, 2012a; Frye, 2012a). Of children with mitochondrial disease and ASD, 41% have seizures (Rossignol and Frye, 2012a).

Other disorders of energy metabolism are associated with ASD and epilepsy, including disorders of creatine metabolism (Póo-Argúelles et al., 2006; Longo et al., 2011) and adenylosuccinate lyase deficiency (Spiegel et al., 2006; Jurecka et al., 2008). Creatine and phosphocreatine play important roles in energy storage and transmission of high-energy phosphates. Adenylosuccinate lyase deficiency is a rare autosomal disorder of *de novo* purine synthesis (Spiegel et al., 2006; Jurecka et al., 2008). The purine nucleotide cycle regulates cellular metabolism by controlling levels of fumarate, a citric acid cycle intermediate, and adenosine, the precursor to adenosine-5′-triphosphate (ATP) (Spiegel et al., 2006).

An energy deficiency can result in seizures. Neurons with high firing rates, such as inhibitory GABA interneurons (Anderson et al., 2008), are disproportionally affected by an energy deficit. In addition, processes critically involved in the release and reuptake of neurotransmitters and maintenance of the neuronal resting potential, such as calcium homeostasis, are critically dependent on mitochondrial function (Li et al., 2004; Quiroz et al., 2008; Chen and Chan, 2009).

#### Disorders of GABA neurotransmission

Several metabolic disorders directly lead to GABA metabolism abnormalities. Pyridoxine and its primary biologically active form, pyridoxal-5-phosphate, are essential cofactors for over 110 enzymes, including glutamic acid decarboxylase (GAD), the enzyme that produces GABA from glutamate. Pyridoxal-5-phosphate depletion reduces GAD activity which, in turn, increases glutamate, decreases GABA synthesis and decreases cortical inhibition (Gospe et al., 1994; Gospe, 2002; Mills et al., 2006). This occurs in pyridoxine dependent seizures.

Succinic semialdehyde dehydrogenase deficiency is an autosomal recessive disorder of GABA metabolism. It results from a defect in the aldehyde dehydrogenase gene (ALDH5A1; Jakobs et al., 1981). Aldehyde dehydrogenase is partially responsible for the degradation of GABA and when this enzyme is deficient GABA is degraded through an alternative pathway, resulting in the formation of gamma-hydroxybutyric acid and GABA elevations in the brain. Positron emission tomography studies suggest that chronic elevation in GABA down-regulates GABA_A_ receptors, leading to a deficit in cortical inhibition and an elevation in the excitatory-to-inhibitory balance (Pearl et al., 2009a,b).

#### Disorders of glutamate neurotransmission

Two types of metabolic disorders (urea cycle defects and phenylketonuria) may result in dysfunction of glutamate neurotransmission. Glutamate is the major excitatory cortical neurotransmitter and excess glutamate results in an elevation in the excitatory-to-inhibitory balance, leading to seizures.

Urea cycle defects result in ammonia elevations. Astrocytes exposed to ammonia do not express glutamate reuptake transporters that normally reduce extracellular glutamate (Rose, 2006). Thus, increased ammonia levels in the brain can result in elevated extracellular glutamate.

Neurological consequences of phenylketonuria are usually avoided by dietary treatment starting at birth (Williams et al., 2008). However, epilepsy and ASD may develop in untreated children and in those noncompliant to the prescribed diet (Baieli et al., 2003). Such children demonstrate high levels of phenylalanine in the brain. Phenylalanine antagonizes both N-methyl-D-aspartate (NMDA) and non-NMDA glutamate receptors (i.e., α-amino-3-hydroxy-5-methyl-4-isoxazolepropionic acid (AMPA) receptors; Glushakov et al., 2003). Chronic elevation in phenylalanine leads to an upregulation of several NMDA and AMPA receptor subunits (Glushakov et al., 2005), increased glutamate receptor density and increased glutamate release (Martynyuk et al., 2005). Such changes in glutamate neurotransmission predispose the brain to heightened excitability and seizures, especially if the phenylalanine level is transiently lowered (Martynyuk et al., 2005).

### Mineral deficiencies

Magnesium is essential in neurotransmitter metabolism and in modulating neurotransmitter receptor function. Ionized magnesium is important in seizure control. Ionized magnesium is a NMDA antagonist (Ault et al., 1980; Hallak, 1998) and may be a factor in some epilepsies (Hallak et al., 1992; Mathern et al., 1998; Mikuni et al., 1999). NMDA receptor activation by glutamate results in calcium influx (Macdermott et al., 1986; Delorenzo and Limbrick, 1996), which is pro-epileptogenic (Delorenzo, 1986; Heinemann and Hamon, 1986). Low ionized magnesium or altered balance between ionized magnesium and ionized calcium may precipitate seizures (Chaistitwanich et al., 1987). Patients with epilepsy have been shown to have significantly lower mean ionized magnesium levels and an increase in the ionized calcium to ionized magnesium ratio in spite of normal total serum magnesium levels (Sinert et al., 2007).

The role of zinc in epilepsy is not clear. Low zinc levels has been associated with seizures in children (Ganesh and Janakiraman, 2008; Mollah et al., 2008) and in the EL epileptic mouse (Fukahori and Itoh, 1990). Zinc acts as an anticonvulsant (Williamson and Spencer, 1995; Cole et al., 2000) and decreases seizure susceptibility (Fukahori and Itoh, 1990). However, zinc has been shown to be proconvulsant in a mouse model (Pei et al., 1983). Zinc co-localizes with glutamate where it inhibits the reuptake of synaptic GABA, thereby increasing the cortical inhibitory tone (Cohen-Kfir et al., 2005). Thus, a zinc deficiency could increase the relative excitatory-to-inhibitory balance.

### Vitamin deficiencies

Children with ASD have been shown to have abnormalities in cobalamin dependent pathways (Frye and James, 2014), and cobalamin supplementation improves metabolites in these pathways (James et al., 2009a; Adams et al., 2011; Frye et al., 2013a; Hendren et al., 2016) and behavior (Adams et al., 2011; Frye et al., 2013a,b; Hendren et al., 2016). The exact mechanism in which cobalamin deficiency causes seizures is unclear but infants with cobalamin deficiency manifest seizures (Benbir et al., 2007; Erol et al., 2007). Cobalamin is essential for myelin synthesis and methylation (Kumar, 2004). Neurons with damaged myelin sheaths are more susceptible to the excitatory effects of glutamate (Akaike et al., 1993).

Cerebral folate deficiency (CFD) is characterized by low 5-methyltetrahydrofolate in the CNS and is associated with ASD and seizures (Ramaekers et al., 2002; Ramaekers and Blau, 2004). Children with idiopathic ASD have a high prevalence of folate receptor alpha autoantibodies that causes CFD (Frye et al., 2013c, 2016b). Folate is essential in a wide range of metabolic processes, including redox and homocysteine metabolism and gene methylation (Obeid et al., 2007). Disruption in these processes could disrupted redox metabolism, thereby depleting glutathione which, in turn, can decreased glutamate degradation, leading to increased cortical excitability (Deepmala et al., 2015).

### Heavy metals

Several epidemiologic studies support a relationship between ASD and exposure to mercury or other heavy metals (Rossignol et al., 2014). Epilepsy has been associated with exposure to toxic levels of heavy metals including lead (Silbergeld et al., 1979; Swartzwelder, 1985; Lockitch et al., 1991; Arrieta et al., 2005) and mercury (Torres et al., 2000). Heavy metals may have toxic effects on the brain by reducing mitochondrial function (James et al., 2009b; Belyaeva et al., 2011; Wang et al., 2011a; Rose et al., 2015), causing apoptosis (Wang et al., 2011a; Pal et al., 2012), and increasing levels of reactive oxygen species (James et al., 2009b; Furieri et al., 2011; Wang et al., 2011a). Although the mechanism(s) by which heavy metals cause epilepsy are not clear, both mitochondrial dysfunction (Rossignol and Frye, 2012a) and high levels of reactive oxygen species (Riazi et al., 2010; Specchio et al., 2010; Waldbaum and Patel, 2010), have been linked to epilepsy.

### Immune dysregulation

Multiple studies have demonstrated evidence of abnormal immune system activation in individuals with ASD. Unusually high levels of proinflammatory cytokines have been found in the cerebrospinal fluid of individuals with ASD (Vargas et al., 2005). Abnormal activation of the intrinsic immune system in the cerebral cortex, white matter, and cerebellum has been demonstrated in individuals with ASD at autopsy (Vargas et al., 2005).

Children with ASD manifest autoantibodies implicated in childhood epilepsy syndromes associated with language regression (Connolly et al., 2006) and cognitive and behavioral changes (Ganor et al., 2004; Vincent et al., 2004) and drug-resistant epilepsy (Majoie et al., 2006) as well as autoantibodies to critical brain elements, such as myelin basic protein, brain derived neurotrophic factor and endothelial cells (Connolly et al., 1999). GAD65 autoantibodies are associated with several neurological disorders including drug-resistant epilepsy (Blanc et al., 2009). One study found GAD65 autoantibodies in 15% of children with ASD (Rout et al., 2012) but other studies have failed to find these autoantibodies in ASD children (Kalra et al., 2015).

Certain autoantibodies, such as the folate receptor alpha autoantibody, could result in specific syndromes like CFD and a recent study suggests that the folate receptor alpha autoantibody may also interfere with cobalamin metabolism (Frye et al., 2016b). Autoantibodies associated with specific seizure syndromes could also result in the dysfunction of specific neural elements. Autoantibodies can also be an epiphenomenon of underlying immune dysregulation.

## Summary

Many of these disorders associated with both seizures and ASD increase the excitatory-to-inhibitory balance. Some disorders reduce brain inhibition by reducing the inhibitory neurotransmitter GABA by a reduction in GABA production, metabolic failure of inhibitory GABA neurons or dysfunction of GABA receptors. Other disorders increase brain excitation by increasing the excitatory neurotransmitter glutamate through increased production, alterations in degradation, or altering glutamate receptors. Independent of these disorders, neuropathological research on ASD points to abnormalities in inhibitory GABA pathways.

A few studies suggest that some gene mutations are associated with a reduction in the excitatory-to-inhibitory balance. This appears to contradict the classic association of seizures with cortical excitability. It may be that these changes cause instability in neuronal networks or compensatory changes at the neuronal level that may create abnormalities in neural excitability. For example, although brain GABA is increased in patients with succinic semialdehyde dehydrogenase deficiency, GABA receptors are down regulated, leading to an elevation in the excitatory-to-inhibitory balance (Pearl et al., 2009a,b).

Clearly further research examining these pathways in more detail could help guide the development of targeted treatments and improve our understanding of the clinical implication of these changes. This review suggests that neurological dysfunction in at least a subset of children with ASD is based on alterations in the excitatory-to inhibitory balance in the brain.

## Author contributions

This manuscript was developed as part of the Elias Tembenis Seizure Think Tanks at the Autism One Meeting in Chicago in May of 2009 and 2010 and at the Autism Canada Meeting in Toronto, Canada in October of 2009. These Think Tanks included scientists and clinicians with expertise in seizures related to ASD. The participants represented a wide variety of researchers and practitioners who treat ASD. Individual participants who provided written text for the supplement or contributed in the editing of the document are recognized as authors. Dr. Fatemi's research fellows assisted with the writing despite not attending the Think Tank so they are recognized as authors.

### Conflict of interest statement

The authors declare that the research was conducted in the absence of any commercial or financial relationships that could be construed as a potential conflict of interest.

## Abbreviations

AMPA: α-amino-3-hydroxy-5-methyl-4-isoxazolepropionic acid
NMDA: N-methyl-D-aspartate
ASD: autism spectrum disorder
ATP: adenosine-5′-triphosphate
CNS: central nervous system
GABA: γ-aminobutyric acid.

## References

[B1] Adams J. B. Audhya T. McDonough-Means S. Rubin R. A. Quig D. Geis E.. (2011). Effect of a vitamin/mineral supplement on children and adults with autism. BMC Pediatr. 11:111. 10.1186/1471-2431-11-111 22151477 PMC3266205

[B2] Akaike A. Tamura Y. Sato Y. Yokota T. (1993). Protective effects of a vitamin B12 analog, methylcobalamin, against glutamate cytotoxicity in cultured cortical neurons. Eur. J. Pharmacol. 241, 1–6. 10.1016/0014-2999(93)90925-8 7901032

[B3] Anderson M. P. Hooker B. S. Herbert M. R. (2008). Bridging from cells to cognition in autism pathophysiology: biological pathways to defective brain function and plasticity Am. J. Biochem. Biotechnol. 4, 167–176. 10.3844/ajbbsp.2008.167.176

[B4] Arrieta O. Palencia G. García-Arenas G. Morales-Espinosa D. Hernández-Pedro N. Sotelo J. (2005). Prolonged exposure to lead lowers the threshold of pentylenetetrazole-induced seizures in rats. Epilepsia 46, 1599–1602. 10.1111/j.1528-1167.2005.00267.x 16190930

[B5] Ault B. Evans R. H. Francis A. A. Oakes D. J. Watkins J. C. (1980). Selective depression of excitatory amino acid induced depolarizations by magnesium ions in isolated spinal cord preparations. J. Physiol. 307, 413–428. 10.1113/jphysiol.1980.sp013443 6259339 PMC1283053

[B6] Baieli S. Pavone L. Meli C. Fiumara A. Coleman M. (2003). Autism and phenylketonuria. J. Autism Dev. Disord. 33, 201–204. 10.1023/A:1022999712639 12757360

[B7] Bangash M. A. Park J. M. Melnikova T. Wang D. Jeon S. K. Lee D.. (2011). Enhanced polyubiquitination of Shank3 and NMDA receptor in a mouse model of autism. Cell 145, 758–772. 10.1016/j.cell.2011.03.052 21565394 PMC3110672

[B8] Belyaeva E. A. Korotkov S. M. Saris N. E. (2011). In vitro modulation of heavy metal-induced rat liver mitochondria dysfunction: a comparison of copper and mercury with cadmium. J. Trace Elem. Med. Biol. 25(Suppl. 1), S63–S73. 10.1016/j.jtemb.2010.10.007 21146384

[B9] Benbir G. Uysal S. Saltik S. Zeybek C. A. Aydin A. Dervent A.. (2007). Seizures during treatment of Vitamin B12 deficiency. Seizure 16, 69–73. 10.1016/j.seizure.2006.10.016 17150378

[B10] Blanc F. Ruppert E. Kleitz C. Valenti M. P. Cretin B. Humbel R. L.. (2009). Acute limbic encephalitis and glutamic acid decarboxylase antibodies: a reality? J. Neurol. Sci. 287, 69–71. 10.1016/j.jns.2009.09.004 19786284

[B11] Blatt G. J. Fitzgerald C. M. Guptill J. T. Booker A. B. Kemper T. L. Bauman M. L. (2001). Density and distribution of hippocampal neurotransmitter receptors in autism: an autoradiographic study. J. Autism Dev. Disord. 31, 537–543. 10.1023/A:1013238809666 11814263

[B12] Brock J. Brown C. C. Boucher J. Rippon G. (2002). The temporal binding deficit hypothesis of autism. Dev. Psychopathol. 14, 209–224. 10.1017/S0954579402002018 12030688

[B13] Brown C. Gruber T. Boucher J. Rippon G. Brock J. (2005). Gamma abnormalities during perception of illusory figures in autism. Cortex 41, 364–376. 10.1016/S0010-9452(08)70273-9 15871601

[B14] Buxhoeveden D. P. Casanova M. F. (2002). The minicolumn hypothesis in neuroscience. Brain 125, 935–951. 10.1093/brain/awf110 11960884

[B15] Casanova M. F. Buxhoeveden D. Gomez J. (2003). Disruption in the inhibitory architecture of the cell minicolumn: implications for autism. Neuroscientist 9, 496–507. 10.1177/1073858403253552 14678582

[B16] Casanova M. Van Kooten I. Switala A. Van Engeland H. Heinsen H. Steinbusch H. (2006). Abnormalities of cortical minicolumnar organization in the prefrontal lobes of autistic patients. Clin. Neurosci. Res. 6, 127–133. 10.1016/j.cnr.2006.06.003

[B17] Chaistitwanich R. Mahoney A. W. Hendricks D. G. Sisson D. V. (1987). Dietary calcium and phosphorus and seizure susceptibility of magnesium deficient rats. Pharmacol. Biochem. Behav. 27, 443–449. 10.1016/0091-3057(87)90347-9 3659067

[B18] Chen H. Chan D. C. (2009). Mitochondrial dynamics–fusion, fission, movement, and mitophagy–in neurodegenerative diseases. Hum. Mol. Genet. 18, R169–R176. 10.1093/hmg/ddp326 19808793 PMC2758711

[B19] Clement J. P. Aceti M. Creson T. K. Ozkan E. D. Shi Y. Reish N. J.. (2012). Pathogenic SYNGAP1 mutations impair cognitive development by disrupting maturation of dendritic spine synapses. Cell 151, 709–723. 10.1016/j.cell.2012.08.045 23141534 PMC3500766

[B20] Cohen-Kfir E. Lee W. Eskandari S. Nelson N. (2005). Zinc inhibition of gamma-aminobutyric acid transporter 4 (GAT4) reveals a link between excitatory and inhibitory neurotransmission. Proc. Natl. Acad. Sci. U.S.A. 102, 6154–6159. 10.1073/pnas.0501431102 15829583 PMC556128

[B21] Cole T. B. Robbins C. A. Wenzel H. J. Schwartzkroin P. A. Palmiter R. D. (2000). Seizures and neuronal damage in mice lacking vesicular zinc. Epilepsy Res. 39, 153–169. 10.1016/S0920-1211(99)00121-7 10759303

[B22] Condie J. Goldstein J. Wainwright M. S. (2010). Acquired microcephaly, regression of milestones, mitochondrial dysfunction, and episodic rigidity in a 46,XY male with a de novo MECP2 gene mutation. J. Child Neurol. 25, 633–636. 10.1177/0883073809342004 20142466

[B23] Connolly A. M. Chez M. G. Pestronk A. Arnold S. T. Mehta S. Deuel R. K. (1999). Serum autoantibodies to brain in Landau-Kleffner variant, autism, and other neurologic disorders. J. Pediatr. 134, 607–613. 10.1016/S0022-3476(99)70248-9 10228297

[B24] Connolly A. M. Chez M. Streif E. M. Keeling R. M. Golumbek P. T. Kwon J. M.. (2006). Brain-derived neurotrophic factor and autoantibodies to neural antigens in sera of children with autistic spectrum disorders, Landau-Kleffner syndrome, and epilepsy. Biol. Psychiatry 59, 354–363. 10.1016/j.biopsych.2005.07.004 16181614

[B25] Danielsson S. Gillberg I. C. Billstedt E. Gillberg C. Olsson I. (2005). Epilepsy in young adults with autism: a prospective population-based follow-up study of 120 individuals diagnosed in childhood. Epilepsia 46, 918–923. 10.1111/j.1528-1167.2005.57504.x 15946331

[B26] Deepmala Slattery, J. Kumar N. Delhey L. Berk M. Dean O.. (2015). Clinical trials of N-acetylcysteine in psychiatry and neurology: a systematic review. Neurosci. Biobehav. Rev. 55, 294–321. 10.1016/j.neubiorev.2015.04.015 25957927

[B27] Defelipe J. (1999). Chandelier cells and epilepsy. Brain 122(Pt 10), 1807–1822. 10.1093/brain/122.10.1807 10506085

[B28] Defelipe J. (2005). Reflections on the structure of the cortical minicolumn, in Neocortical Modularity and the Cell Minicolumn, ed Casanova M. F. (New York, NY: Nova Biomedical), 57–92.

[B29] Defelipe J. Hendry S. H. Hashikawa T. Molinari M. Jones E. G. (1990). A microcolumnar structure of monkey cerebral cortex revealed by immunocytochemical studies of double bouquet cell axons. Neuroscience 37, 655–673. 10.1016/0306-4522(90)90097-N 1701039

[B30] Delgado-Escueta A. V. (2007). Advances in genetics of juvenile myoclonic epilepsies. Epilepsy Curr. 7, 61–67. 10.1111/j.1535-7511.2007.00171.x 17520076 PMC1874323

[B31] Delorenzo R. J. (1986). A molecular approach to the calcium signal in brain: relationship to synaptic modulation and seizure discharge. Adv. Neurol. 44, 435–464. 3010680

[B32] Delorenzo R. J. Limbrick D. D. Jr. (1996). Effects of glutamate on calcium influx and sequestration/extrusion mechanisms in hippocampal neurons. Adv. Neurol. 71, 37–46. 8790787

[B33] Deykin E. Y. Macmahon B. (1979). The incidence of seizures among children with autistic symptoms. Am. J. Psychiatry 136, 1310–1312. 10.1176/ajp.136.10.1310 484727

[B34] Dhossche D. Applegate H. Abraham A. Maertens P. Bland L. Bencsath A.. (2002). Elevated plasma gamma-aminobutyric acid (GABA) levels in autistic youngsters: stimulus for a GABA hypothesis of autism. Med. Sci. Monit. 8, PR1–PR6. 12165753

[B35] D'hulst C. Kooy R. F. (2007). The GABAA receptor: a novel target for treatment of fragile X? Trends Neurosci. 30, 425–431. 10.1016/j.tins.2007.06.003 17590448

[B36] Erol I. Alehan F. Gümüs A. (2007). West syndrome in an infant with vitamin B12 deficiency in the absence of macrocytic anaemia. Dev. Med. Child Neurol. 49, 774–776. 10.1111/j.1469-8749.2007.00774.x 17880648

[B37] Fatemi S. H. (2008). The hyperglutamatergic hypothesis of autism. Prog. Neuropsychopharmacol. Biol. Psychiatry. 32, 911. author reply: 912–913. 18160196 10.1016/j.pnpbp.2007.11.004

[B38] Fatemi S. H. Reutiman T. J. Folsom T. D. Thuras P. D. (2009). GABA(A) receptor downregulation in brains of subjects with autism. J. Autism Dev. Disord. 39, 223–230. 10.1007/s10803-008-0646-7 18821008 PMC2697059

[B39] Favorov O. V. Kelly D. G. (1994). Minicolumnar organization within somatosensory cortical segregates: II. Emergent functional properties. Cereb. Cortex 4, 428–442. 10.1093/cercor/4.4.428 7950313

[B40] Frye R. E. (2012a). Biomarker of abnormal energy metabolism in children with autism spectrum disorder. North Am. J. Med. Sci. 5, 141–147. 10.7156/v5i3p141

[B41] Frye R. E. (2012b). Mitochondrial disease in 22q13 duplication syndrome. J. Child Neurol. 27, 942–949. 10.1177/0883073811429858 22378673

[B42] Frye R. E. (2012c). Novel mitochondrial Cytochrome B gene polymorphisms associated with autism. J. Pediatr. Neurol. 10, 35–40. 10.3233/JPN-2012-0530

[B43] Frye R. E. (2014). Clinical potential, safety, and tolerability of arbaclofen in the treatment of autism spectrum disorder. Drug Healthc. Patient Saf. 6, 69–76. 10.2147/DHPS.S39595 24872724 PMC4025936

[B44] Frye R. E. (2015). Metabolic and mitochondrial disorders associated with epilepsy in children with autism spectrum disorder. Epilepsy Behav. 47, 147–157. 10.1016/j.yebeh.2014.08.134 25440829

[B45] Frye R. E. Cox D. Slattery J. Tippett M. Kahler S. Granpeesheh D.. (2016a). Mitochondrial Dysfunction may explain symptom variation in Phelan-McDermid Syndrome. Sci. Rep. 6, 19544. 10.1038/srep19544 26822410 PMC4731780

[B46] Frye R. E. Delatorre R. Taylor H. B. Slattery J. Melnyk S. Chowdhury N.. (2013a). Metabolic effects of sapropterin treatment in autism spectrum disorder: a preliminary study. Transl. Psychiatry 3:e237. 10.1038/tp.2013.14 23462988 PMC3625913

[B47] Frye R. E. Delhey L. Slattery J. Tippett M. Wynne R. Rose S.. (2016b). Blocking and binding folate receptor alpha autoantibodies identify novel autism spectrum disorder subgroups. Front. Neurosci. 10:80. 10.3389/fnins.2016.00080 27013943 PMC4783401

[B48] Frye R. E. James S. J. (2014). Metabolic pathology of autism in relation to redox metabolism. Biomark. Med. 8, 321–330. 10.2217/bmm.13.158 24712422

[B49] Frye R. E. Melnyk S. Fuchs G. Reid T. Jernigan S. Pavliv O.. (2013b). Effectiveness of methylcobalamin and folinic Acid treatment on adaptive behavior in children with autistic disorder is related to glutathione redox status. Autism Res. Treat. 2013, 609705. 10.1155/2013/609705 24224089 PMC3810468

[B50] Frye R. E. Naviaux R. K. (2011). Autistic disorder with complex IV overactivity: a new mitochondrial syndrome. J. Pediatr. Neurol. 9, 427–434. 10.3233/JPN-2011-0507

[B51] Frye R. E. Rossignol D. A. (2011). Mitochondrial dysfunction can connect the diverse medical symptoms associated with autism spectrum disorders. Pediatr. Res. 69, 41R–47R. 10.1203/pdr.0b013e318212f16b 21289536 PMC3179978

[B52] Frye R. E. Sequeira J. M. Quadros E. V. James S. J. Rossignol D. A. (2013c). Cerebral folate receptor autoantibodies in autism spectrum disorder. Mol. Psychiatry 18, 369–381. 10.1038/mp.2011.175 22230883 PMC3578948

[B53] Fukahori M. Itoh M. (1990). Effects of dietary zinc status on seizure susceptibility and hippocampal zinc content in the El (epilepsy) mouse. Brain Res. 529, 16–22. 10.1016/0006-8993(90)90806-M 2282491

[B54] Furieri L. B. Galán M. Avendaño M. S. García-Redondo A. B. Aguado A. Martínez S.. (2011). Endothelial dysfunction of rat coronary arteries after exposure to low concentrations of mercury is dependent on reactive oxygen species. Br. J. Pharmacol. 162, 1819–1831. 10.1111/j.1476-5381.2011.01203.x 21232032 PMC3081124

[B55] Ganesh R. Janakiraman L. (2008). Serum zinc levels in children with simple febrile seizure. Clin. Pediatr. 47, 164–166. 10.1177/0009922807306165 17873242

[B56] Ganor Y. Goldberg-Stern H. Amrom D. Lerman-Sagie T. Teichberg V. I. Pelled D.. (2004). Autoimmune epilepsy: some epilepsy patients harbor autoantibodies to glutamate receptors and dsDNA on both sides of the blood-brain barrier, which may kill neurons and decrease in brain fluids after hemispherotomy. Clin. Dev. Immunol. 11, 241–252. 10.1080/17402520400001736 15559370 PMC2486323

[B57] Giulivi C. Zhang Y. F. Omanska-Klusek A. Ross-Inta C. Wong S. Hertz-Picciotto I.. (2010). Mitochondrial dysfunction in autism. JAMA 304, 2389–2396. 10.1001/jama.2010.1706 21119085 PMC3915058

[B58] Glushakov A. V. Dennis D. M. Sumners C. Seubert C. N. Martynyuk A. E. (2003). L-phenylalanine selectively depresses currents at glutamatergic excitatory synapses. J. Neurosci. Res. 72, 116–124. 10.1002/jnr.10569 12645085

[B59] Glushakov A. V. Glushakova O. Varshney M. Bajpai L. K. Sumners C. Laipis P. J.. (2005). Long-term changes in glutamatergic synaptic transmission in phenylketonuria. Brain 128, 300–307. 10.1093/brain/awh354 15634735

[B60] Gospe S. M. (2002). Pyridoxine-dependent seizures: findings from recent studies pose new questions. Pediatr. Neurol. 26, 181–185. 10.1016/S0887-8994(01)00407-6 11955923

[B61] Gospe S. M. Jr. Olin K. L. Keen C. L. (1994). Reduced GABA synthesis in pyridoxine-dependent seizures. Lancet 343, 1133–1134. 10.1016/S0140-6736(94)90236-4 7910233

[B62] Grice S. J. Spratling M. W. Karmiloff-Smith A. Halit H. Csibra G. De Haan M.. (2001). Disordered visual processing and oscillatory brain activity in autism and Williams syndrome. Neuroreport 12, 2697–2700. 10.1097/00001756-200108280-00021 11522950

[B63] Grothe B. Klump G. M. (2000). Temporal processing in sensory systems. Curr. Opin. Neurobiol. 10, 467–473. 10.1016/S0959-4388(00)00115-X 10981615

[B64] Hallak M. (1998). Effect of parenteral magnesium sulfate administration on excitatory amino acid receptors in the rat brain. Magnes. Res. 11, 117–131. 9675756

[B65] Hallak M. Berman R. F. Irtenkauf S. M. Evans M. I. Cotton D. B. (1992). Peripheral magnesium sulfate enters the brain and increases the threshold for hippocampal seizures in rats. Am. J. Obstet. Gynecol. 167, 1605–1610. 10.1016/0002-9378(92)91749-Z 1471674

[B66] Han Y. Qin J. Bu D. F. Chang X. Z. Yang Z. X. (2006). Successive alterations of hippocampal gamma-aminobutyric acid B receptor subunits in a rat model of febrile seizure. Life Sci. 78, 2944–2952. 10.1016/j.lfs.2005.11.023 16380138

[B67] Hara H. (2007). Autism and epilepsy: a retrospective follow-up study. Brain Dev. 29, 486–490. 10.1016/j.braindev.2006.12.012 17321709

[B68] Heinemann U. Hamon B. (1986). Calcium and epileptogenesis. Exp. Brain Res. 65, 1–10. 10.1007/BF00243826 3026832

[B69] Hendren R. L. James S. J. Widjaja F. Lawton B. Rosenblatt A. Bent S. (2016). Randomized, placebo-controlled trial of Methyl B12 for children with autism. J. Child Adolesc. Psychopharmacol. 10.1089/cap.2015.0159. [Epub ahead of print]. 26889605

[B70] Jakobs C. Bojasch M. Mönch E. Rating D. Siemes H. Hanefeld F. (1981). Urinary excretion of gamma-hydroxybutyric acid in a patient with neurological abnormalities. The probability of a new inborn error of metabolism. Clin. Chim. Acta 111, 169–178. 10.1016/0009-8981(81)90184-4 7226548

[B71] James S. J. Melnyk S. Fuchs G. Reid T. Jernigan S. Pavliv O.. (2009a). Efficacy of methylcobalamin and folinic acid treatment on glutathione redox status in children with autism. Am. J. Clin. Nutr. 89, 425–430. 10.3945/ajcn.2008.26615 19056591 PMC2647708

[B72] James S. J. Rose S. Melnyk S. Jernigan S. Blossom S. Pavliv O.. (2009b). Cellular and mitochondrial glutathione redox imbalance in lymphoblastoid cells derived from children with autism. FASEB J. 23, 2374–2383. 10.1096/fj.08-128926 19307255 PMC2717775

[B73] Jurecka A. Zikanova M. Tylki-Szymanska A. Krijt J. Bogdanska A. Gradowska W.. (2008). Clinical, biochemical and molecular findings in seven Polish patients with adenylosuccinate lyase deficiency. Mol. Genet. Metab. 94, 435–442. 10.1016/j.ymgme.2008.04.013 18524658

[B74] Kalra S. Burbelo P. D. Bayat A. Ching K. H. Thurm A. Iadarola M. J.. (2015). No evidence of antibodies against GAD65 and other specific antigens in children with autism. BBA Clin. 4, 81–84. 10.1016/j.bbacli.2015.08.001 26366376 PMC4564997

[B75] Kang J. Q. Shen W. Macdonald R. L. (2009). Two molecular pathways (NMD and ERAD) contribute to a genetic epilepsy associated with the GABA(A) receptor GABRA1 PTC mutation, 975delC, S326fs328X. J. Neurosci. 29, 2833–2844. 10.1523/JNEUROSCI.4512-08.2009 19261879 PMC2687144

[B76] Karayannis T. Au E. Patel J. C. Kruglikov I. Markx S. Delorme R.. (2014). Cntnap4 differentially contributes to GABAergic and dopaminergic synaptic transmission. Nature 511, 236–240. 10.1038/nature13248 24870235 PMC4281262

[B77] Kriaucionis S. Paterson A. Curtis J. Guy J. Macleod N. Bird A. (2006). Gene expression analysis exposes mitochondrial abnormalities in a mouse model of Rett syndrome. Mol. Cell. Biol. 26, 5033–5042. 10.1128/MCB.01665-05 16782889 PMC1489175

[B78] Kumar S. (2004). Recurrent seizures: an unusual manifestation of vitamin B12 deficiency. Neurol. India 52, 122–123. 15069260

[B79] Li L. Chin L. S. Shupliakov O. Brodin L. Sihra T. S. Hvalby O.. (1995). Impairment of synaptic vesicle clustering and of synaptic transmission, and increased seizure propensity, in synapsin I-deficient mice. Proc. Natl. Acad. Sci. U.S.A. 92, 9235–9239. 10.1073/pnas.92.20.9235 7568108 PMC40959

[B80] Li Z. Okamoto K. Hayashi Y. Sheng M. (2004). The importance of dendritic mitochondria in the morphogenesis and plasticity of spines and synapses. Cell 119, 873–887. 10.1016/j.cell.2004.11.003 15607982

[B81] Lockitch G. Berry B. Roland E. Wadsworth L. Kaikov Y. Mirhady F. (1991). Seizures in a 10-week-old infant: lead poisoning from an unexpected source. CMAJ 145, 1465–1468. 1959106 PMC1336037

[B82] Longo N. Ardon O. Vanzo R. Schwartz E. Pasquali M. (2011). Disorders of creatine transport and metabolism. Am. J. Med. Genet. C Semin. Med. Genet. 157C, 72–78. 10.1002/ajmg.c.30292 21308988

[B83] Macdermott A. B. Mayer M. L. Westbrook G. L. Smith S. J. Barker J. L. (1986). NMDA-receptor activation increases cytoplasmic calcium concentration in cultured spinal cord neurones. Nature 321, 519–522. 10.1038/321519a0 3012362

[B84] Majoie H. J. De Baets M. Renier W. Lang B. Vincent A. (2006). Antibodies to voltage-gated potassium and calcium channels in epilepsy. Epilepsy Res. 71, 135–141. 10.1016/j.eplepsyres.2006.06.003 16870397

[B85] Martynyuk A. E. Glushakov A. V. Sumners C. Laipis P. J. Dennis D. M. Seubert C. N. (2005). Impaired glutamatergic synaptic transmission in the PKU brain. Mol. Genet. Metab. 86(Suppl. 1), S34–S42. 10.1016/j.ymgme.2005.06.014 16153867

[B86] Mathern G. W. Pretorius J. K. Mendoza D. Lozada A. Leite J. P. Chimelli L.. (1998). Increased hippocampal AMPA and NMDA receptor subunit immunoreactivity in temporal lobe epilepsy patients. J. Neuropathol. Exp. Neurol. 57, 615–634. 10.1097/00005072-199806000-00008 9630240

[B87] Mikuni N. Babb T. L. Ying Z. Najm I. Nishiyama K. Wylie C.. (1999). NMDA-receptors 1 and 2A/B coassembly increased in human epileptic focal cortical dysplasia. Epilepsia 40, 1683–1687. 10.1111/j.1528-1157.1999.tb01584.x 10612330

[B88] Mills P. B. Struys E. Jakobs C. Plecko B. Baxter P. Baumgartner M.. (2006). Mutations in antiquitin in individuals with pyridoxine-dependent seizures. Nat. Med. 12, 307–309. 10.1038/nm1366 16491085

[B89] Mollah M. A. Rakshit S. C. Anwar K. S. Arslan M. I. Saha N. Ahmed S.. (2008). Zinc concentration in serum and cerebrospinal fluid simultaneously decrease in children with febrile seizure: findings from a prospective study in Bangladesh. Acta Paediatr. 97, 1707–1711. 10.1111/j.1651-2227.2008.01001.x 18795906

[B90] Mountcastle V. B. (1997). The columnar organization of the neocortex. Brain 120(Pt 4), 701–722. 10.1093/brain/120.4.701 9153131

[B91] Murdoch J. D. State M. W. (2013). Recent developments in the genetics of autism spectrum disorders. Curr. Opin. Genet. Dev. 23, 310–315. 10.1016/j.gde.2013.02.003 23537858

[B92] Obeid R. Mccaddon A. Herrmann W. (2007). The role of hyperhomocysteinemia and B-vitamin deficiency in neurological and psychiatric diseases. Clin. Chem. Lab. Med. 45, 1590–1606. 10.1515/CCLM.2007.356 18067446

[B93] Pal P. B. Pal S. Das J. Sil P. C. (2012). Modulation of mercury-induced mitochondria-dependent apoptosis by glycine in hepatocytes. Amino Acids 42, 1669–1683. 10.1007/s00726-011-0869-3 21373768

[B94] Pearl P. L. Gibson K. M. Cortez M. A. Wu Y. Carter Snead O. 3rd. Knerr I.. (2009a). Succinic semialdehyde dehydrogenase deficiency: lessons from mice and men. J. Inherit. Metab. Dis. 32, 343–352. 10.1007/s10545-009-1034-y 19172412 PMC2693236

[B95] Pearl P. L. Gibson K. M. Quezado Z. Dustin I. Taylor J. Trzcinski S.. (2009b). Decreased GABA-A binding on FMZ-PET in succinic semialdehyde dehydrogenase deficiency. Neurology 73, 423–429. 10.1212/WNL.0b013e3181b163a5 19667317 PMC2727143

[B96] Pei Y. Zhao D. Huang J. Cao L. (1983). Zinc-induced seizures: a new experimental model of epilepsy. Epilepsia 24, 169–176. 10.1111/j.1528-1157.1983.tb04876.x 6832078

[B97] Pelc K. Boyd S. G. Cheron G. Dan B. (2008). Epilepsy in Angelman syndrome. Seizure 17, 211–217. 10.1016/j.seizure.2007.08.004 17904873

[B98] Peñagarikano O. Abrahams B. S. Herman E. I. Winden K. D. Gdalyahu A. Dong H.. (2011). Absence of CNTNAP2 leads to epilepsy, neuronal migration abnormalities, and core autism-related deficits. Cell 147, 235–246. 10.1016/j.cell.2011.08.040 21962519 PMC3390029

[B99] Pickett J. Xiu E. Tuchman R. Dawson G. Lajonchere C. (2011). Mortality in individuals with autism, with and without epilepsy. J. Child Neurol. 26, 932–939. 10.1177/0883073811402203 21471551

[B100] Póo-Argüelles P. Arias A. Vilaseca M. A. Ribes A. Artuch R. Sans-Fito A.. (2006). X-Linked creatine transporter deficiency in two patients with severe mental retardation and autism. J. Inherit. Metab. Dis. 29, 220–223. 10.1007/s10545-006-0212-4 16601898

[B101] Princivalle A. P. Richards D. A. Duncan J. S. Spreafico R. Bowery N. G. (2003). Modification of GABA(B1) and GABA(B2) receptor subunits in the somatosensory cerebral cortex and thalamus of rats with absence seizures (GAERS). Epilepsy Res. 55, 39–51. 10.1016/S0920-1211(03)00090-1 12948615

[B102] Quiroz J. A. Gray N. A. Kato T. Manji H. K. (2008). Mitochondrially mediated plasticity in the pathophysiology and treatment of bipolar disorder. Neuropsychopharmacology 33, 2551–2565. 10.1038/sj.npp.1301671 18235426

[B103] Ramaekers V. T. Blau N. (2004). Cerebral folate deficiency. Dev. Med. Child Neurol. 46, 843–851. 10.1111/j.1469-8749.2004.tb00451.x 15581159

[B104] Ramaekers V. T. Häusler M. Opladen T. Heimann G. Blau N. (2002). Psychomotor retardation, spastic paraplegia, cerebellar ataxia and dyskinesia associated with low 5-methyltetrahydrofolate in cerebrospinal fluid: a novel neurometabolic condition responding to folinic acid substitution. Neuropediatrics 33, 301–308. 10.1055/s-2002-37082 12571785

[B105] Riazi K. Galic M. A. Pittman Q. J. (2010). Contributions of peripheral inflammation to seizure susceptibility: cytokines and brain excitability. Epilepsy Res. 89, 34–42. 10.1016/j.eplepsyres.2009.09.004 19804959

[B106] Rippon G. Brock J. Brown C. Boucher J. (2007). Disordered connectivity in the autistic brain: challenges for the ‘new psychophysiology’. Int. J. Psychophysiol. 63, 164–172. 10.1016/j.ijpsycho.2006.03.012 16820239

[B107] Rose C. (2006). Effect of ammonia on astrocytic glutamate uptake/release mechanisms. J. Neurochem. 97(Suppl. 1), 11–15. 10.1111/j.1471-4159.2006.03796.x 16635245

[B108] Rose S. Frye R. E. Slattery J. Wynne R. Tippett M. Melnyk S.. (2014a). Oxidative stress induces mitochondrial dysfunction in a subset of autistic lymphoblastoid cell lines. Transl. Psychiatry 4:e377. 10.1038/tp.2014.15 24690598 PMC4012280

[B109] Rose S. Frye R. E. Slattery J. Wynne R. Tippett M. Pavliv O.. (2014b). Oxidative stress induces mitochondrial dysfunction in a subset of autism lymphoblastoid cell lines in a well-matched case control cohort. PLoS ONE 9:e85436. 10.1371/journal.pone.0085436 24416410 PMC3885720

[B110] Rose S. Wynne R. Frye R. E. Melnyk S. James S. J. (2015). Increased susceptibility to ethylmercury-induced mitochondrial dysfunction in a subset of autism lymphoblastoid cell lines. J. Toxicol. 2015, 573701. 10.1155/2015/573701 25688267 PMC4320799

[B111] Rossignol D. A. Frye R. E. (2012a). Mitochondrial dysfunction in autism spectrum disorders: a systematic review and meta-analysis. Mol. Psychiatry 17, 290–314. 10.1038/mp.2010.136 21263444 PMC3285768

[B112] Rossignol D. A. Frye R. E. (2012b). A review of research trends in physiological abnormalities in autism spectrum disorders: immune dysregulation, inflammation, oxidative stress, mitochondrial dysfunction and environmental toxicant exposures. Mol. Psychiatry 17, 389–401. 10.1038/mp.2011.165 22143005 PMC3317062

[B113] Rossignol D. A. Genuis S. J. Frye R. E. (2014). Environmental toxicants and autism spectrum disorders: a systematic review. Transl. Psychiatry 4:e360. 10.1038/tp.2014.4 24518398 PMC3944636

[B114] Rout U. K. Mungan N. K. Dhossche D. M. (2012). Presence of GAD65 autoantibodies in the serum of children with autism or ADHD. Eur. Child Adolesc. Psychiatry 21, 141–147. 10.1007/s00787-012-0245-1 22323074

[B115] Rubenstein J. L. Merzenich M. M. (2003). Model of autism: increased ratio of excitation/inhibition in key neural systems. Genes Brain Behav. 2, 255–267. 10.1034/j.1601-183X.2003.00037.x 14606691 PMC6748642

[B116] Sansa G. Carlson C. Doyle W. Weiner H. L. Bluvstein J. Barr W.. (2011). Medically refractory epilepsy in autism. Epilepsia 52, 1071–1075. 10.1111/j.1528-1167.2011.03069.x 21671922

[B117] Schaefer G. B. Mendelsohn N. J. Professional P. Guidelines C. (2013). Clinical genetics evaluation in identifying the etiology of autism spectrum disorders: 2013 guideline revisions. Genet. Med. 15, 399–407. 10.1038/gim.2013.32 23519317

[B118] Schuler V. Lüscher C. Blanchet C. Klíx N. Sansig G. Klebs K.. (2001). Epilepsy, hyperalgesia, impaired memory, and loss of pre- and postsynaptic GABA(B) responses in mice lacking GABA(B(1)). Neuron 31, 47–58. 10.1016/S0896-6273(01)00345-2 11498050

[B119] Shavelle R. M. Strauss D. J. Pickett J. (2001). Causes of death in autism. J. Autism Dev. Disord. 31, 569–576. 10.1023/A:1013247011483 11814268

[B120] Shcheglovitov A. Shcheglovitova O. Yazawa M. Portmann T. Shu R. Sebastiano V.. (2013). SHANK3 and IGF1 restore synaptic deficits in neurons from 22q13 deletion syndrome patients. Nature 503, 267–271. 10.1038/nature12618 24132240 PMC5559273

[B121] Silbergeld E. K. Miller L. P. Kennedy S. Eng N. (1979). Lead, GABA, and seizures: effects of subencephalopathic lead exposure on seizure sensitivity and GABAergic function. Environ. Res. 19, 371–382. 10.1016/0013-9351(79)90062-8 227673

[B122] Sinert R. Zehtabchi S. Desai S. Peacock P. Altura B. T. Altura B. M. (2007). Serum ionized magnesium and calcium levels in adult patients with seizures. Scand. J. Clin. Lab. Invest. 67, 317–326. 10.1080/00365510601051441 17454846

[B123] Specchio N. Fusco L. Claps D. Vigevano F. (2010). Epileptic encephalopathy in children possibly related to immune-mediated pathogenesis. Brain Dev. 32, 51–56. 10.1016/j.braindev.2009.09.017 19850427

[B124] Spiegel E. K. Colman R. F. Patterson D. (2006). Adenylosuccinate lyase deficiency. Mol. Genet. Metab. 89, 19–31. 10.1016/j.ymgme.2006.04.018 16839792

[B125] Srivastava A. K. Schwartz C. E. (2014). Intellectual disability and autism spectrum disorders: causal genes and molecular mechanisms. Neurosci. Biobehav. Rev. 46(Pt 2), 161–174. 10.1016/j.neubiorev.2014.02.015 24709068 PMC4185273

[B126] Su H. Fan W. Coskun P. E. Vesa J. Gold J. A. Jiang Y. H.. (2011). Mitochondrial dysfunction in CA1 hippocampal neurons of the UBE3A deficient mouse model for Angelman syndrome. Neurosci. Lett. 487, 129–133. 10.1016/j.neulet.2009.06.079 19563863 PMC2888840

[B127] Swartzwelder H. S. (1985). Central neurotoxicity after exposure to organic lead: susceptibility to seizures. Neurosci. Lett. 58, 225–228. 10.1016/0304-3940(85)90168-5 4047483

[B128] Tabuchi K. Blundell J. Etherton M. R. Hammer R. E. Liu X. Powell C. M.. (2007). A neuroligin-3 mutation implicated in autism increases inhibitory synaptic transmission in mice. Science 318, 71–76. 10.1126/science.1146221 17823315 PMC3235367

[B129] Tommerdahl M. Tannan V. Cascio C. J. Baranek G. T. Whitsel B. L. (2007). Vibrotactile adaptation fails to enhance spatial localization in adults with autism. Brain Res. 1154, 116–123. 10.1016/j.brainres.2007.04.032 17498672 PMC1987714

[B130] Torres A. D. Rai A. N. Hardiek M. L. (2000). Mercury intoxication and arterial hypertension: report of two patients and review of the literature. Pediatrics 105:E34. 10.1542/peds.105.3.e34 10699136

[B131] Tuchman R. (2013). Autism and social cognition in epilepsy: implications for comprehensive epilepsy care. Curr. Opin. Neurol. 26, 214–218. 10.1097/WCO.0b013e32835ee64f 23400237

[B132] Tuchman R. F. Rapin I. (1997). Regression in pervasive developmental disorders: seizures and epileptiform electroencephalogram correlates. Pediatrics 99, 560–566. 10.1542/peds.99.4.560 9093299

[B133] Tuchman R. Hirtz D. Mamounas L. A. (2013). NINDS epilepsy and autism spectrum disorders workshop report. Neurology 81, 1630–1636. 10.1212/WNL.0b013e3182a9f482 24089385 PMC3806917

[B134] Tuchman R. Rapin I. (2002). Epilepsy in autism. Lancet Neurol. 1, 352–358. 10.1016/S1474-4422(02)00160-6 12849396

[B135] Vargas D. L. Nascimbene C. Krishnan C. Zimmerman A. W. Pardo C. A. (2005). Neuroglial activation and neuroinflammation in the brain of patients with autism. Ann. Neurol. 57, 67–81. 10.1002/ana.20315 15546155

[B136] Vincent A. Buckley C. Schott J. M. Baker I. Dewar B. K. Detert N.. (2004). Potassium channel antibody-associated encephalopathy: a potentially immunotherapy-responsive form of limbic encephalitis. Brain 127, 701–712. 10.1093/brain/awh077 14960497

[B137] Volkmar F. R. Nelson D. S. (1990). Seizure disorders in autism. J. Am. Acad. Child Adolesc. Psychiatry 29, 127–129. 10.1097/00004583-199001000-00020 2295565

[B138] Waldbaum S. Patel M. (2010). Mitochondria, oxidative stress, and temporal lobe epilepsy. Epilepsy Res. 88, 23–45. 10.1016/j.eplepsyres.2009.09.020 19850449 PMC3236664

[B139] Wang L. Wang H. Li J. Chen D. Liu Z. (2011a). Simultaneous effects of lead and cadmium on primary cultures of rat proximal tubular cells: interaction of apoptosis and oxidative stress. Arch. Environ. Contam. Toxicol. 61, 500–511. 10.1007/s00244-011-9644-4 21287161

[B140] Wang X. Mccoy P. A. Rodriguiz R. M. Pan Y. Je H. S. Roberts A. C.. (2011b). Synaptic dysfunction and abnormal behaviors in mice lacking major isoforms of Shank3. Hum. Mol. Genet. 20, 3093–3108. 10.1093/hmg/ddr212 21558424 PMC3131048

[B141] Williams R. A. Mamotte C. D. Burnett J. R. (2008). Phenylketonuria: an inborn error of phenylalanine metabolism. Clin. Biochem. Rev. 29, 31–41. 18566668 PMC2423317

[B142] Williamson A. Spencer D. (1995). Zinc reduces dentate granule cell hyperexcitability in epileptic humans. Neuroreport 6, 1562–1564. 10.1097/00001756-199507310-00024 7579149

[B143] Zablotsky B. Black L. I. Maenner M. J. Schieve L. A. Blumberg S. J. (2015). Estimated Prevalence of Autism and Other Developmental Disabilities Following Questionnaire Changes in the 2014 National Health Interview Survey. Hyattsville, MD: National Center for Health Statistics. National Health Statistics Reports No. 87. 26632847

